# Shading as a Control Method for Invasive European Frogbit (*Hydrocharis morsus-ranae* L.)

**DOI:** 10.1371/journal.pone.0098488

**Published:** 2014-06-02

**Authors:** Bin Zhu, Michael S. Ellis, Kelly L. Fancher, Lars G. Rudstam

**Affiliations:** 1 Department of Biology, University of Hartford, West Hartford, Connecticut, United States of America; 2 Department of Biology, Hobart and William Smith Colleges, Geneva, New York, United States of America; 3 Department of Natural Resources, Cornell University, Ithaca, New York, United States of America; North Carolina State University, United States of America

## Abstract

Invasive European frogbit (*Hydrocharis morsus-ranae* L.) has negative environmental and economic impacts in North American water bodies. It is therefore important to develop effective management tools to control this invasive species. This study investigated shading as a control method for European frogbit in both greenhouse and lake mesocosm experiments. A series of shade treatments (0%, 50%, 60%, 70%, 80%, and 100%) were tested in the greenhouse for three weeks. Results showed that the 100% shade was most effective at controlling European frogbit, and other shade treatments greater than 50% were less effective, reducing frogbit biomass up to 38.2%. There were no differences found in temperature between treatments, but dissolved oxygen decreased as shading increased. A lake mesocosm experiment utilizing 0% shade, 70% shade, and 100% shade treatments was performed in a sheltered inlet of Oneida Lake in New York State for over one month. Resulting European frogbit biomass was significantly (25 times) less in areas treated with the 70% shade and nearly zero with the 100% shade. Shading did not affect temperature but improved DO conditions. Results on the shading effects on submerged macrophytes were not conclusive: no significant differences in changes in species richness and abundance between the three groups at the end of studied period suggested no shading effects; significant differences between the beginning and end communities in the 70% shade and the 100% shade but not in the control group indicated significant impacts of shading. This study is the first one to investigate shading as a control method for European frogbit and it is concluded that a moderately high density shade can effective remove European frogbit likely with minor impacts on the environment. More experiments with larger scales and longer time periods are recommended for further investigation.

## Introduction

Biological, physical, and chemical factors all interact to shape the growth, abundance, and distribution of aquatic plants [1], [2], [3]. Important factors affecting aquatic plants include light, depth, fetch, ice scour, latitude, temperature, and water levels. Among these factors, light is of paramount importance, because it exerts a major control on photosynthesis and declines with water depth due to attenuation, scattering, and absorption [4], [5]. The general effect of an increase in light will be positive for aquatic plants, promoting plant growth by increasing photosynthesis [6], [7]. For example, Zhu et al. [8] reported the submerged macrophytes increased their abundance, richness and depths in Oneida Lake, NY when the water clarity increased following the invasion of zebra mussels and nutrient reduction. Extension of submerged plant distribution to deeper depth was observed in bays of the Great Lakes as the water clarity increased [2], [9]. Conversely, blocking light will lead to reduction in plant growth [5], [6], [10]. Therefore, shading as a control method is likely to inhibit growth of invasive aquatic plants.

European frogbit (*Hydrocharis morsus-ranae* L.) is an invasive floating plant in North American water bodies. The species escaped in 1939 from a Botanical Garden in Ottawa, Canada and was then spotted in the Rideau Canal, Ontario, Canada [11]. European frogbit has been travelling south since then and had reached the United States by 1974 [12]. This plant can be found in still, slow-moving shallow waters, such as ponds, ditches, wetlands, marshes and swamps, backwaters, beaver dams, canals, and sluggish creeks, as well as wind sheltered and wave protected areas of lakes and rivers [13], [14]. European frogbit reproduces vegetatively through development of stolon buds and turions to form new plantlets and possibly through seeds as well [14]. It continues to spread and may invade further south as a result of range expansion. Global warming may facilitate spreading of this species [15]. European frogbit's dense leaves cover a large surface area that blocks or reduces sunlight penetrating the water below, thereby suppressing growth of submerged, native macrophytes [16]. Invasive European frogbit may harm the economy as well as the environment because of its ability to expand rapidly. It can block navigation channels, irrigation ditches, and water intake pipes and can reduce aesthetic and recreational value of water bodies, thus decreasing tourism and real estate values [14], [17]. It is therefore important to develop effective management tools to control this invasive species.

Mechanical harvesting, chemicals, and biological agents are three common methods suggested or reported to control European frogbit. While a mechanical harvesting technique is a control method, it can have significant negative impacts on aquatic ecosystems [14]. Hand pulling has also been proven helpful in removing some frogbit from numerous environments [18]. However, this approach regularly requires the employment of costly labor forces [18] and frequent, repeated removal efforts in order to be effective (B. Zhu, unpublished data). Chemicals such as endothal and diquat have been used as effective controls in ditches against European frogbit [19], [20]. However, chemical treatment sometimes is not target-specific and can eliminate other aquatic plants, including beneficial species, and possibly have negative impacts on other organisms [20]. Biological control agents were also suggested in some studies because European frogbit is a food resource for many animals including insects, rodents, water birds, freshwater snails, and fish [14], [21], [22], [23]. Froemming [21] observed that consumption of *H. morsus-ranae* stimulates egg production of the freshwater snails *Lymnaea stagnalis* and *Rumina decollate*. Dabbling ducks (*Anas* spp.) have been documented to consume European frogbit in the eutrophic wetlands of central Finland [22]. Note though, these biological control candidates are not target-specific and could harm other native plants or animals [22]. To date, there are no classical biological control organisms in development or released for European frogbit.

Studies have shown that light is essential for the germination and growth of European frogbit, that light-deprivation may reduce frogbit root growth by 90% [24], [25]. It follows that control methods utilizing shading are likely to inhibit the growth and spread of this invasive species. Shading has already been successfully used for controlling aquatic plants such as submerged cabomba (*Cabomba caroliniana*) [10]. It was reported that the 99% shade completely removed cabomba within four months and the 70% shade was effective at deeper depths [10]. However, shading may have negative impacts on beneficial submerged plants that grow underneath target species due to further reductions in the amount of light penetrating to deeper depths [16]. Therefore, shading that blocks too much light (e.g., >90%) would not be desirable despite its high potential to eradicate target species. A desirable outcome should result in the effective control of European frogbit and minor or no impacts on submerged macrophytes below. Consequently, the objectives of this study are: 1) to test the efficacy of different levels of shading as controls on European frogbit growth in both greenhouse and lake mesocosm experiments; 2) to assess the impacts of the shading method on aquatic ecosystems by examining temperature, dissolved oxygen, and submerged macrophytes underneath treated European frogbit mats.

## Materials and Methods

### Shading Experiment in the Greenhouse

An experiment with a random design was performed in a greenhouse using black shade cloths of various densities (50%, 60%, 70%, 80%, and 100%, International Greenhouse Company, Georgetown, IL) for three weeks from June 1 to June 21, 2010. We also included ambient light (0% shade) as the control. The greenhouse had air exchange with the outside and the temperature and irradiance were similar to ambient lake conditions. Individual full-grown plantlets were put in 5 gallon white buckets with different shade cloths (see Zhu et al. [15] for details), and all plantlets were similar in size at the beginning of the experiment. All buckets were placed in one large water bath at the ambient temperature to simulate lake conditions - the shallow portion of a lake where European frogbit is likely to grow. Three replicates were randomly selected for each shade level. Temperature and dissolved oxygen (DO) were recorded at the end of the experiment using an Orion 4 Star DO Portable multipurpose digital probe (Thermo Scientific, Waltham, MA). European frogbit was collected at the end and biomass was weighed after drying at 65°C for 72 hrs. Plant growth was evaluated by the number of plantlets in each bucket, the average biomass per plantlet, and total biomass at the end of the experiment.

### Shading Experiment in the Lake Mesocosms

The natural ecosystem testing was performed in a sheltered inlet in Big Bay of Oneida Lake in New York State (43.25^o^ N, 76.11^o^ W) from June 23 to July 27, 2010. No specific permissions were required for this location for the purpose of this experiment and our field study did not involve endangered or protected species. A control group (European frogbit mats with no shading), 70% shade, and 100% shade were applied to 1×1 m^2^ experiment plots with similar densities (about 80% coverage) of European frogbit, three replicates for each group. Experiment plots were defined by 1 m×1 m PVC pipe squares set floating on the surface, anchored with concrete blocks to the bottom of the lake, and labeled and kept afloat with buoys. Water depth in each plot was less than 1.5 m, and all plots were located in areas protected from wind and waves and with minimal boat traffic. The 70% shade treatment was chosen mainly based on the results from the greenhouse experiment (see the result section for details). Also in another study, the 70% shade was used to control submerged cabomba [10]. Therefore, we chose the 70% shade instead of a series of shade gradients in the lake experiment. Temperature and DO were measured below the cloths at noon of three separated dates (June 23, July 8, and July 27) during the experiment period. Total biomass of European frogbit was collected and measured at the end of the experiment to test the effectiveness of the shade method. Submerged aquatic macrophytes were collected at the beginning and the end of this experiment using a 0.25 m^2^ (0.5 m×0.5 m) quadrat below each plot to evaluate the impacts of the shade method on submerged macrophytes. Dry weight was measured after drying at 65°C for 72 hrs and biomass was then calculated for analysis.

### Statistical Analysis

All data except submerged macrophyte biomass were recorded as mean ±1 standard error. Standard errors were not shown for submerged macrophytes due to large variability of pre-existing submerged macrophyte communities between experimental sites within each group. We used non-metric multidimensional scaling (MDS) [26] to visualize differences in plant community structure before and after the experimental treatment. The differences among plots were based on the biomass (dry weight) of nine species of macrophytes. Significant differences between community structure before the experiment and community structure in the control group, the 70% shade and 100% shade treatments (four factors) were investigated with an analysis of similarities (ANOSIM) [26]. ANOSIM used all possible permutations of the rank similarity matrix to calculate the probability of the similarity within a factor to be larger than a random selection of samples using an R-statistics defined as the difference between the average of rank similarities of pairs of plots in different groups and the average of rank similarities of pairs of plots within a group, and dividing this difference by a measure of the number of samples under consideration [26]. Analyses were done with Primer v6.1.6 (Plymouth Routines in Multivariate Ecological Research). For other comparisons, all data was natural logarithm transformed (ln(x+1)) to reduce heteroscedasticity and analyzed using ANOVA (IBM SPSS Statistic 20) [27]. All ANOVAs were followed by the least significant difference (LSD) analysis to compare different treatments at the level α = 0.05 [27].

## Results

### Greenhouse Experiment

#### Effectiveness of Shading

**Figure 1:**
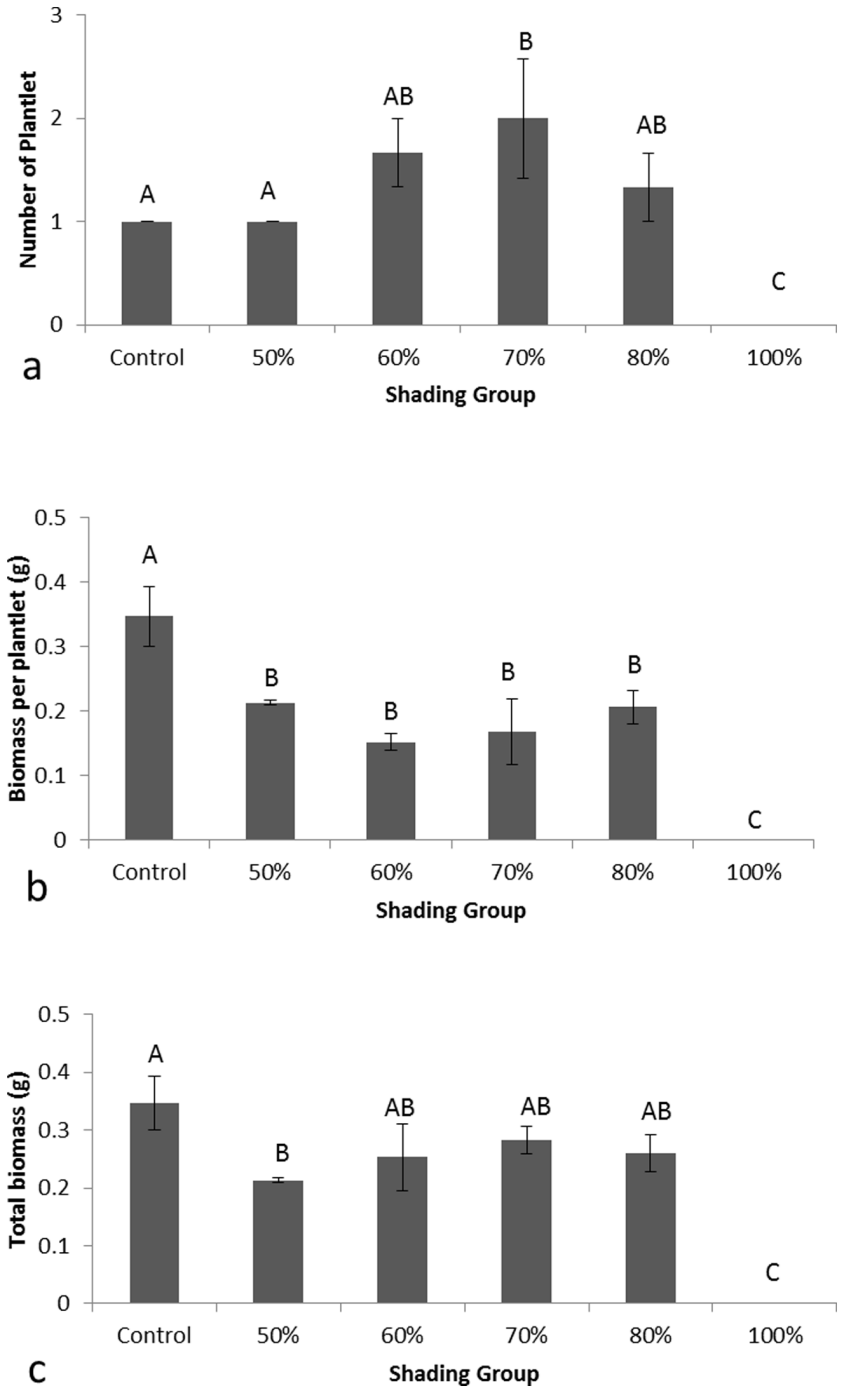
European frogbit growth in a series of shades in the greenhouse experiment: a. number of plantlets, b. average biomass per plantlet, and c. total biomass. Different letters indicate significant differences at α = 0.05 following ANOVA LSD analysis.

Plant growth was evaluated from three different variables: number of plantlets, average biomass per plantlet, and total biomass for all the plantlets in each group. We observed that the control group had the healthiest plants while no plants survived after the 100% shade treatment, and all treatments with 50% shading or higher affected frogbit growth (Figure 1). The control group with ambient light had an average of one plantlet after three weeks, as did the 50% shade treatment, while all other shading treatments but the 100% shade had more plantlets than the control group (Figure 1a). Average biomass per plantlet was highest in the control group with 0.35±0.05 g/plantlet while all shaded treatments were significantly lower (Figure 1b). The lowest was observed in the 100% shade treatment followed by the 60% and 70% shade groups. Like average biomass per plantlet, the treatment groups had much lower total biomass than the control group, with zero biomass under the 100% shade (Figure 1c). Combining the results from the three variables, we concluded that 100% shade was most effective for controlling European frogbit. Other shade treatments greater than 50% were relatively effective, reducing the biomass up to 38.2%.

#### Impacts of Shading on Temperature and DO

**Figure 2:**
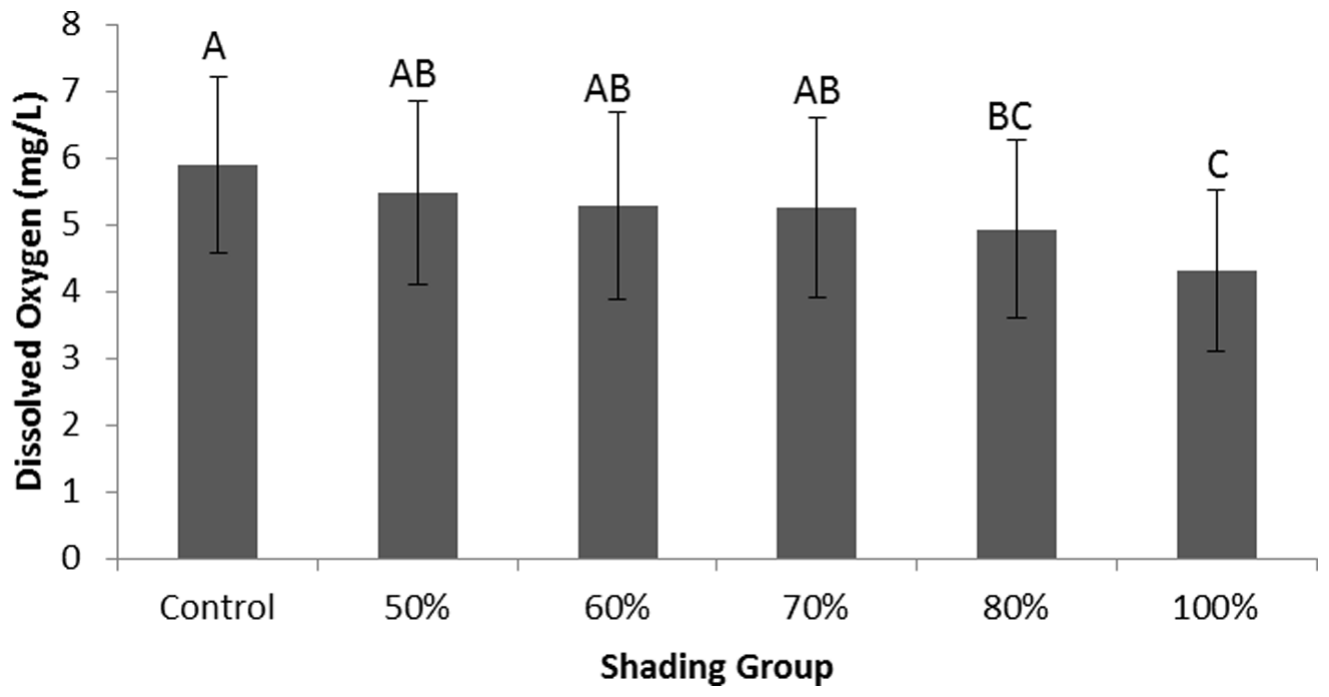
Dissolved oxygen in each shade treatment in the greenhouse experiment. Different letters indicate significant differences at α = 0.05 following ANOVA LSD analysis.

Water temperature under different shade treatments ranged from 21.4±0.44°C to 22.7±0.15°C and were not statistically different (df = 5, F = 2.077, p = 0.139). However, there were differences in dissolved oxygen content between the different groups (df = 5, F = 3.783, p = 0.027, Figure 2). Dissolved oxygen decreased when more light was blocked, from 5.9±1.3 mg/L in the control group to 4.3±1.2 mg/L in the 100% shade.

### Lake Mesocosm Experiment

#### Effectiveness of Shading

Data from the lake mesocosm experiment suggested shading had effectively controlled European frogbit: its biomass was significantly reduced from 142.6±9.6 g/m^2^ in the control group to 5.7±3.5 g/m^2^ in the 70% shade, and to 0.01±0.01 g/m^2^ in the 100% shade (df = 2, F = 174.9, p<0.001).

#### Impacts of Shading on Temperature and DO

**Figure 3:**
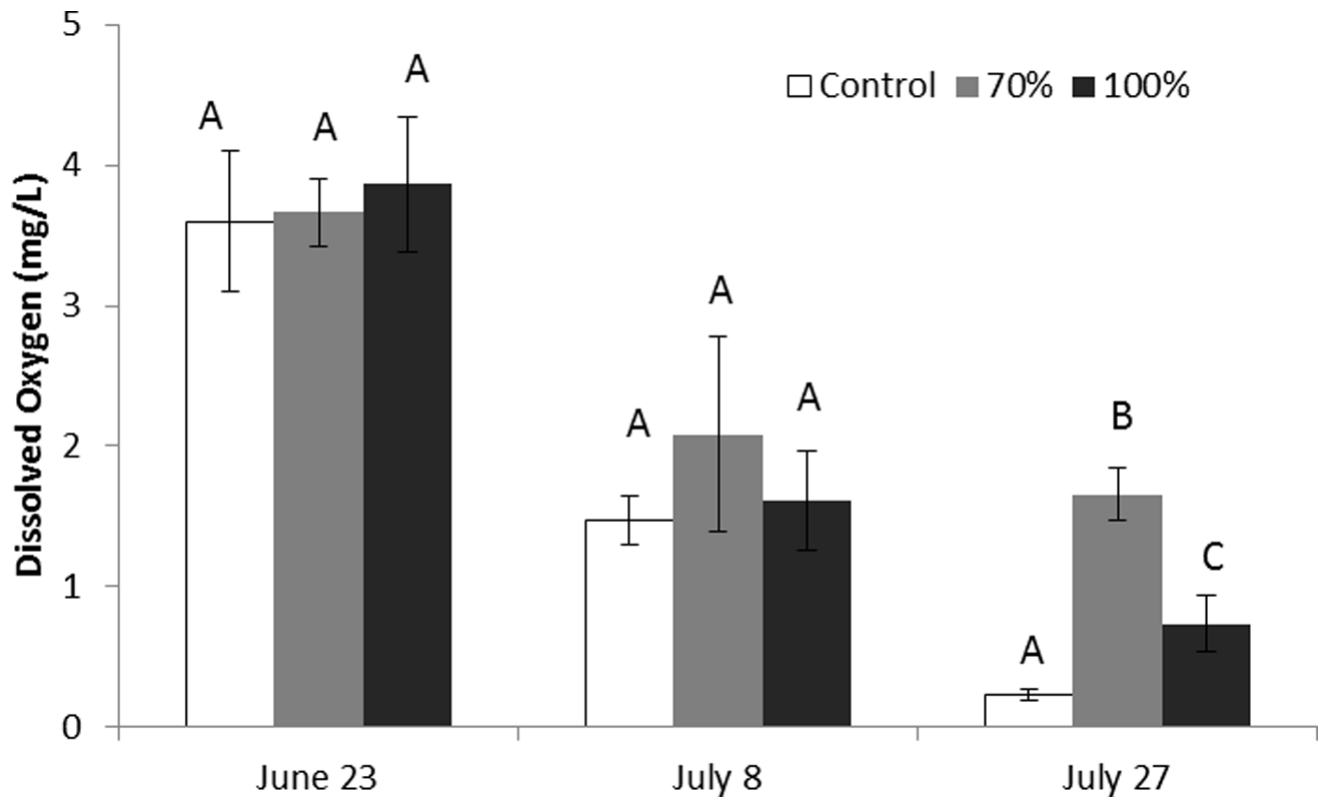
Dissolved oxygen in different treatment groups at different times during the lake mesocosm experiment. Different letters indicate significant differences at α = 0.05 following ANOVA LSD analysis.

There was an obvious seasonal trend in temperature (df = 2, F = 40.2, p<0.001), but no differences were found between the three different groups: control, 70% shade, or 100% shade in the lake (df = 2, F = 0.564, p = 0.576). DO levels were generally low (less than 4 mg/L) in densely vegetated water in this study (Figure 3). As the experiment proceeded, DO decreased from around 4 mg/L to less than 1.5 mg/L. However more oxygen was present in the two shade groups than the control group at the end of the experiment (1.66±0.19 and 0.74±0.20 mg/L vs. 0.23±0.04 mg/L, df = 2, F = 20.57, p = 0.002, Figure 3).

#### Effects of Shading on Submerged Macrophytes

Species richness of submerged macrophytes was found not significantly different between the control group and the shading treatment groups at the beginning of the experiments (df = 2, F = 1.091, p = 0.394) or at the end of the experiments (df = 2, F = 1.50, p = 0.296, Table 1). Species richness was similar for all treatments at the beginning of the experiment and had declined by the end for all treatments, including the control. Relatively more reduction in macrophyte richness occurred in plots with the 100% shade compared to those in the control group and under the 70% shade (Table 1).

**Table 1:** Species richness and biomass of submerged macrophytes in different shading treatments.

Shade Group	Species Richness			Biomass (g/m^2^)	
	Start	End	Start		End
Control	7.0±0.6	6.0±0.6	33.2 (25.5, 43.6)		8.5 (1.2, 13.8)
70%	8.3±0.9	5.3±0.9	40.3 (15.8, 88.8)		17.5 (2.8, 45.1)
100%	7.7±0.3	3.7±1.3	31.5 (10.7, 62.8)		1.8 (0.4, 3.8)

Species richness was shown as the mean ±1 SE and biomass was shown as the mean followed by the range of biomass in parenthesis.

**Figure 4:**
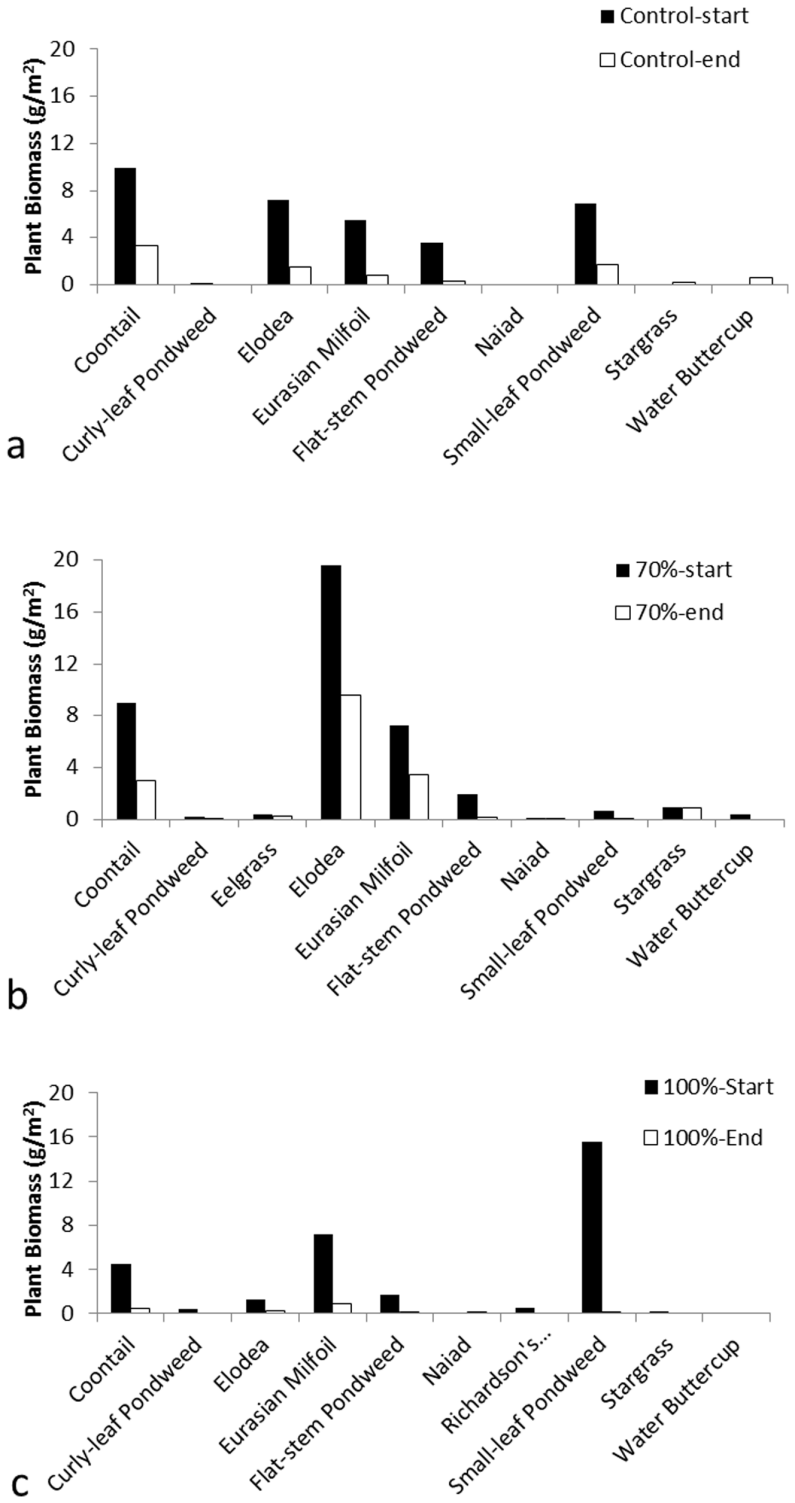
The species composition and abundance of submerged macrophytes at the start and the end of the lake experiment: a. the control group where European frogbit mats were present; b. the 70% shade; and c. the 100% shade. Due to large variability of pre-existing submerged macrophyte communities between experimental sites within each group, only means were shown here and standard errors were omitted for clear view.

Results of the impacts of shading on the submerged macrophyte community stucture in terms of species composition and abundance were not conclusive. The species composition varied among groups (Figure 4). For all groups, the dominant submerged macrophyte species were coontail (*Ceratophyllum demersum*), elodea (*Elodea canadensis*), Eurasian watermilfoil (*Myriophyllum spicatum*), flat-stem pondweed (*Potamogeton zosteriformis*), and small-leaf pondweed (*P. pusillus*), though their abundance (in terms of biomass) differed between groups (Figure 4). For example, at the beginning of the experiment, coontail was the most abundant species in the control group whereas elodea was most abundant in the 70% shade, and small-leaf pondweed was most abundant in the 100% shade. This also indicated the variability of initial submerged macrophyte communities. At the end of the experiment, most species had decreased in biomass, but the magnitude of this change varied between groups. Seven out of the nine species in the control group had 65.9%–100% (average of 85.4%) reduction in biomass whereas two species, stargrass (*Heteranthera dubia*) and water buttercup (*Ranunculus aquatilis*), increased in biomass depsite their overall abundance being relatively low. Similarly, eight out of the ten species in the 70% shade experienced 45.2%–100% (average of 68.6%) reduction in biomass, and two species, common naiad (*Najas flexilis*) and stargrass, slightly increased in biomass. However, the densities of all ten species were reduced by 57.1%–100% (average of 90.6%, Figure 4) in plots treated with the 100% shade. These changes led to the decrease in total abundance of submerged macrophyte (measured as biomass of all macrophytes) in all the three groups: 69.6% in the control group, 67.8% in the 70% shade group, and 89.8% in the 100% shade group (Table 1) and the changes did not differ between groups (df = 2, F = 1.288, p = 0.342). There were also no differences in the community structures at end of the experiment among the three groups (R<0.01, p>0.55). However, significant differences were found when the beginning and end community structures were compared for the three groups. There were no differences in the beginning and end community structures in the control group (R = 0.36, p = 0.077) whereas significant differences between the beginning and end communities were found in the 70% shade and the 100% shade (R = 0.45, p = 0.03, and R = 0.84, p = 0.005 respectively).

## Discussion

Our results from both greenhouse and lake experiments revealed that the 100% shade was most effective at removing invasive European frogbit. However this level of shading should be considered unacceptable as a large-scale control option since its application significantly reduces native plant growth and would be detrimental to many other aquatic organisms [28].

A moderately high density of shade, such as 70%, did effectively control invasive European frogbit and could be a control option. In our lake mesocosm experiment, the 70% shade significantly reduced frogbit biomass by 25 times over the course of a one month trial, while in the greenhouse experiment, shade treatments greater than 50% were all relatively effective in the control of European frogbit, especially in reducing frogbit biomass. Interestingly, greater reproduction (indicated by the number of plantlets) was observed in the denser shade treatments. Because plants often show greater vegetative growth at the expense of reproduction under favorable conditions [29], this change in resource allocation from growth to reproduction is a predictable response to the low-light stress created by denser shading. For example, Miller et al. [29] reported that dioecious hydrilla (*Hydrilla verticillata*) decreased turion production when plant density increased (i.e., better growth). In stressful situations where survival probability is low and opportunities for reproduction may be limited to “now or never,” plants tend to assign more resources to reproductive output [30]. However, if the stress persists, affected plants will eventually die.

Control or eradication efforts including physical, chemical and/or biological methods, often greatly disturb natural ecosystems, possibly increasing the vulnerability of an area to subsequent invaders [3], [28]. An effective control method should be able to remove unwanted plants while minimizing environmental impacts. Shading had no impacts on temperature in the growing environment and may be able to improve DO condition, as was demonstrated by the 70% shade treatments in our lake mesocosm experiment, which had higher DO values than both the control and 100% shade treatments. Higher DO is likely improving environmental conditions for organisms living beneath the plants, including fish and benthic invertebrates. The lowest DO content was observed in the lake control group at the end of the experiment and may have been attributable to the control group's dense, floating mats of European frogbit cover (142.6 g/m^2^ compared to 5.7 g/m^2^ in the 70% shade). Conversely, in the greenhouse experiment, DO content was highest in the control group, which was likely a result of there being only one plant in each bucket, with plenty of gas exchange between air and water. DO content in the greenhouse experiment decreased as shade increased, likely because there was less photosynthesis under shade and greater rates of decomposition resulted from increased frogbit mortality alongside shade density. These same reasons also explain the low final DO observed in the 100% shade lake treatments. Highest DO in the 70% shade in the lake experiment may be due to less decomposition and more algal production after the European frogbit canopy was removed [31].

The lake experiment suggested possible negative impacts of the shade treatments on submerged macrophytes growing underneath the European frogbit mats. Like the control group, there was an overall decline in total macrophyte biomass along with a slight change in species richness in both the 70% shade and the 100% shade. However, there were no differences in the beginning and the end community structures in the control group whereas significant differences between the beginning and end communities were found in the 70% shade and 100% shading. This indicates there were likely significant impacts of shading on submerged macrophyte communities. Typically we would expect high levels of shading would have negative impacts on species richness and abundance of submerged plants because light is a key factor regulating submerged plant growth and depth distribution, both of which decrease as light is reduced [1], [2], [5], [10]. Other factors might have weakened the negative effects of shading in this study such as large variation of pre-existing macrophyte communities and relatively small size of shading plots in the trial. Because the shading plot is small, submerged macrophytes can still have some light from the surrounding areas, which minimizes the shading effects. Therefore, experiments further investigating the effects of shading over larger spatial scales and longer time periods are recommended 1) because decreases in submerged macrophyte biomass were observed under all treatments in this study, 2) because a possible seasonal reduction in macrophyte biomass cannot be eliminated as a factor, and 3) because of the variability of pre-existing macrophyte communities between sites within the different shade groups at the beginning of the experiment.

Our lake mesocosm and greenhouse experiments have demonstrated that a moderately high density of shading, such as that achieved with the 70% shade cloth, can serve as an effective control for European frogbit. In some conditions, it may even improve some envrionmental conditions such as dissolved oxygen compared to the environment under dense beds of European frogbit. However, this might also have some negative impacts on submerged macrophytes. Shading has not been regularly used for controlling aquatic plants to date, with few invasive aquatic plant management cases utilizing this method [10]. The major reason is that shades are thought to interfere with recreation and are considered aesthetically displeasing because shades float on the water [32]. However, shading may be a feasible choice for controlling European frogbit because it grows in sheltered areas where there are few recreational activities. Additionally, shading has desirable qualities for invasive plant management – time and cost efficient. Shading requires only the preparation, placement, and retrieval of shade cloths, and the shade cloth is commercially available at low prices. The success and the feasibility of eradication are usually heavily dependent upon the amount of investment that can be made [33]. Consequently, it seems both effective and feasible to use shading to control European frogbit.

There are other options for controlling European frogbit, including manual or mechanical removal of plants, chemical control, and biological control [18], [20], [22]. All control mechanisms, including the shading method, have advantages and disadvantages. For example, shading can be effective, but it may affect other floating plants growing next to European frogbit. It is noteworthy to mention that prevention is the single best solution for European frogbit management, just as it is for many other invasive species [17], [28], [32]. Common methods of prevention include decontamination and cleaning of boats and equipment that could contain hitchhikers and restricting deliberate imports of potentially harmful species. However, once European frogbit is established, shading may be considered by aquatic plant managers as one of the possible control methods.

## References

[1] Spence DHN (1982) The zonation of plants in freshwater lakes. Adv Ecol Res 12: 37–125.

[2] Skubinna JP, Coon TG, Batterson TR (1995) Increased abundance and depth of submersed macrophytes in response to decreased turbidity in Saginaw Bay, Lake Huron. J Great Lakes Res 21: 476–488.

[3] Schindler DE, Scheuerell MD (2002) Habitat coupling in lake ecosystems. Oikos 98: 177–189.

[4] Sand-Jensen K, Borum J (1991) Interactions among phytoplankton, periphyton, and macrophytes in temperate freshwaters and estuaries. Aquat Bot 41: 137–175.

[5] Hudon C, Lalonde S, Gagnon P (2000) Ranking the effects of site exposure, plant growth form, water depth, and transparency on aquatic plant biomass. Can J Fish Aquat Sci (Suppl 1):31–42.

[6] Wade PM (1990) Physical control of aquatic weeds. In: Pieterse AH, Murphy KJ, editors. Aquatic Weeds: The Ecology and Management of Nuisance Aquatic Vegetation. New York: Oxford University Press. pp. 93–135.

[7] Karatayev AY, Burlakova LE, Padilla K (2002) Impacts of zebra mussels on aquatic communities and their roles as ecosystem engineers. In: Leppakoski E, Ollasch S, Olenin S, editors. Invasive aquatic species of Europe: distribution, impacts and management. Boston: Kluwer. pp. 433–446.

[8] Zhu B, Fitzgerald DG, Mayer CM, Rudstam LG, Mills EL (2006) Alteration of ecosystem function by zebra mussels in Oneida Lake, NY: impacts on submerged macrophytes. Ecosystems 9: 1017–1028.

[9] Zhu B, Fitzgerald DG, Hoskins SB, Rudstam LG, Mayer CM, et al (2007) Quantification of historical changes of submerged aquatic vegetation cover in two bays of Lake Ontario with three complementary methods. J Great Lakes Res 33: 122–135.

[10] Schooler SS (2008) Shade as a management tool for the invasive submerged macrophyte, *Cabomba caroliniana* . J Aquat Plant Manage 46: 168–171.

[11] Dore WG (1968) Progress of the European frogbit in Canada. Can Field-Nat 82: 76–84.

[12] Roberts ML, Stuckey RL, Mitchell RS (1981) *Hydrocharis morsus-ranae* (Hydrocharitaceae): new to the United States. Rhodora 83: 147–148.

[13] Cook CDK, Lüönd R (1982) A revision of the genus Hydrocharis (Hydrocharitaceae). Aquat Bot 14: 177–204.

[14] Catling PM, Miltrow G, Haber E, Posluszny U, Charlton WA (2003) The biology of Canadian weeds. 124. *Hydrocharis morsus-ranae* L. Can J Plant Sci 83: 1001–1016.

[15] Zhu B, Eppers ME, Rudstam LG (2008) Predicting invasion of European frogbit in the Finger Lakes of New York. J Aquat Plant Manage 46: 186–189.

[16] Catling PM, Spicer KW, Lefkovitch LP (1988) Effects of the floating *Hydrocharis morsus-ranae* (Hydrocharitaceae), on some North American aquatic macrophytes. Nat Can 115: 131–137.

[17] Pimentel D, Zuniga R, Morrison D (2005) Update on the environmental and economic costs associated with alien-invasive species in the United States. Ecol Econ 52: 273–288.

[18] Langdon S (2007) Eradication of European Frogbit (*Hydrocharis morsus-ranae*) from the Grasse River in the Town of Clare, St. Lawrence County, New York. Adirondack Park Invasive Plant Program 2007 Season Report. 23 p.

[19] Holz W (1963) Chemical weed control in ditches; trials on the control of submerged plants. 5. Dtsch. Arbeitsbesprechung uber Fragen der Unkrautbiologie u. -bekampfung, Hohenheim, 4 p.

[20] Renard C (1963) The use of diquat and paraquat to control aquatic plants. Compte rendu Conference du Comite Francais de Lutte contre les Mauvaises Herbes (COLUMA). 9 p.

[21] Froemming E (1954) Problematic constituents in several marsh plants. Pharmazi 9: 766–769. 13236499

[22] Vaananen VM, Nummi P (2003) Diet of sympatric dabbling ducks in eutrophic wetlands. Suomen Riista 49: 7–16.

[23] Magomaev FM (1973) The daily diet of two- and three-year-old grass carp. Sbornik Nauchnykh Trudov, Vsesoyuznyi Nauchno-Issledovatel'skii Institut Prudovogo Rybnogo Khozyaistva 10: 192–196.

[24] Minshall WH (1959) Effect of light on the extension growth of roots of frog-bit. Can J Bot 37: 1134–1136.

[25] Richards AJ, Blakemore J (1975) Factors affecting the germination of turions in *Hydrocharis morsus-ranae* L. Watsonia. 10: 273–275.

[26] Clarke KR, Warwick RM (2001) Change in marine communities: an approach to statistical analysis and interpretation. Primer-E Ltd. Plymouth, UK.

[27] Kuehl RO (2000) Design of experiments: statistical principles of research design and analysis. 2nd edition. Pacific Grove, CA: Duxbury Press. 666 p.

[28] Madsen JD (1997) Methods for management of nonindigenous aquatic plants. In: Luken JO, Thieret JW, editors. Assessment and Management of Plant Invasions. Springer, NY. pp.145–171.

[29] Miller JD, Haller WT, Glenn MS (1993) Turion production by dioecious hydrilla in North Florida. J Aquat Plant Manage 31: 101–105.

[30] Chiariello NR, Gulmon SL (1991) Stress effects on plant reproduction. In: Winner WE, Pell EJ, Roy J, editors. Response of Plants to Multiple Stresses. New York: Academic Press. pp. 161–188.

[31] Reed DC, Foster MS (1984) The effects of canopy shading on algal recruitment and growth in a giant kelp forest. Ecology 65: 937–948.

[32] New York State Department of Environmental Conservation (2005) A Primer on Aquatic Plant Management in New York State. Division of Water. 63 p.

[33] Panetta FD (2009) Weed eradication - an economic perspective. Invasive Plant Sci Manage 2: 360–368.

